# pH Drives Differences in Bacterial Community β-Diversity in Hydrologically Connected Lake Sediments

**DOI:** 10.3390/microorganisms11030676

**Published:** 2023-03-07

**Authors:** Haiguang Pu, Yuxiang Yuan, Lei Qin, Xiaohui Liu

**Affiliations:** 1 Key Laboratory of Wetland Ecology and Environment, Northeast Institute of Geography and Agroecology, Chinese Academy of Sciences, Changchun 130102, China; 2 University of Chinese Academy of Sciences, Beijing 101408, China

**Keywords:** sediment, community structure and diversity, microbial assembly, environmental variables, Xingkai Lake

## Abstract

As microorganisms are very sensitive to changes in the lake environment, a comprehensive and systematic understanding of the structure and diversity of lake sediment microbial communities can provide feedback on sediment status and lake ecosystem protection. Xiao Xingkai Lake (XXL) and Xingkai Lake (XL) are two neighboring lakes hydrologically connected by a gate and dam, with extensive agricultural practices and other human activities existing in the surrounding area. In view of this, we selected XXL and XL as the study area and divided the area into three regions (XXLR, XXLD, and XLD) according to different hydrological conditions. We investigated the physicochemical properties of surface sediments in different regions and the structure and diversity of bacterial communities using high-throughput sequencing. The results showed that various nutrients (nitrogen, phosphorus) and carbon (DOC, LOC, TC) were significantly enriched in the XXLD region. Proteobacteria, Firmicutes, and Bacteroidetes were the dominant bacterial phyla in the sediments, accounting for more than 60% of the entire community in all regions. Non-metric multidimensional scaling analysis and analysis of similarities confirmed that β-diversity varied among different regions. In addition, the assembly of bacterial communities was dominated by a heterogeneous selection in different regions, indicating the important influence of sediment environmental factors on the community. Among these sediment properties, the partial least squares path analysis revealed that pH was the best predictor variable driving differences in bacterial communities in different regions, with higher pH reducing beta diversity among communities. Overall, our study focused on the structure and diversity of bacterial communities in lake sediments of the Xingkai Lake basin and revealed that high pH causes the β-diversity of bacterial communities in the sediment to decrease. This provides a reference for further studies on sediment microorganisms in the Xingkai Lake basin in the future.

## 1. Introduction

As one of the important components of global aquatic ecosystems, lakes play key roles in global hydrological processes, biodiversity maintenance, and biogeochemical cycles owing to their ability to process nutrients over long hydraulic retention times [1,2,3]. Although lakes barely occupy 3.7% of the global land surface, they globally distribute and collect considerable deposit substance from local basins, resulting in rich material, information, and intense energy exchanges [4,5,6]. In this context, lakes have become a research hotspot in the field of biogeochemistry. Sediments are an important part of lake ecosystems, considered to be sinks or sources for nutrient cycling, and are among the most varied microbial habitats in the aquatic environment [7]. Sediments play an essential role in substance-circulating in the system, including numerous transformation and migration processes, such as adsorption–desorption, migration and dissolution, deposition [8], as well as biodegradation and bioaccumulation at the soil–water interface [9,10]. Additionally, sediment aerobic and anaerobic environments are more complex. Organisms depend on substances containing heavy metal ions, organic matter, and animal and plant remains to maintain their biochemical activities in sediments [11].

Lake sediments, which have been demonstrated to be major reservoirs of carbon and other materials, involve high biomass and microbial taxa richness [12]. Sediment-attached microbes serve as an essential driver of lake biogeochemical processes regulating the majority of biogeochemical cycles and nutrient transformations [13]. As sediment bacterial communities are very susceptible to chemical and physical variations, their structures may consequently respond to environmental changes spatially and temporally [14]. As a result, their richness, diversity, and physiological activity may be affected, and even the function of some bacteria may be inhibited. Therefore, more and more studies focus on these environmental regulation factors causing sediment microbial changes, including sediment texture, nutrient load, and sediment pH [3,15,16].

Xingkai Lake is an important boundary lake between China and Russia, containing various ecological functions. There are many wetlands, swamps, and farmlands in the Xingkai Lake basin. Rapid shrinkage and fragmentation have occurred in the Xingkai Lake Basin in the past fifty years [17]. Furthermore, nutrient concentrations have increased and water quality has decreased in the basin due to the constant use of fertilizers in agricultural activities. A previous study has reported that about 7.6 t/a TN and 5.4 t/a TP input into the basin [18]. Due to the interference of agricultural pollution and other human activities, the original ecological balance of Xiao Xingkai Lake has been broken, and in the water body, severe eutrophication with sediment that was seriously polluted has appeared. Although less affected by human activities, the water body of Xingkai Lake has experienced mesotrophic-eutrophic due to its hydrological connection with Xiao Xingkai Lake. Harmful algae blooms, which frequently occur in Xiao Xingkai Lake, flood into Xingkai Lake. There have been many studies on the sediments of Xiao Xingkai Lake and Xingkai Lake, including the carbon cycle and the temporal and spatial changes of heavy metals [19,20]. However, few studies have investigated the sediment bacterial community structure and diversity in the area.

In our study, we divided the study area into three regions according to the special topography of Xiao Xingkai Lake and Xingkai Lake. Our objectives were: (1) to investigate the physical and chemical characteristics of sediments in different regions; (2) to explore the community assembly processes and the community structure and diversity; (3) to determine the factors driving bacterial community structure and diversity. Our research can provide a scientific basis for the restoration and development of the lake ecosystem of Xingkai Lake in the future.

## 2. Materials and Methods

### 2.1. Sample Sites and Sample Collections

**Figure 1:**
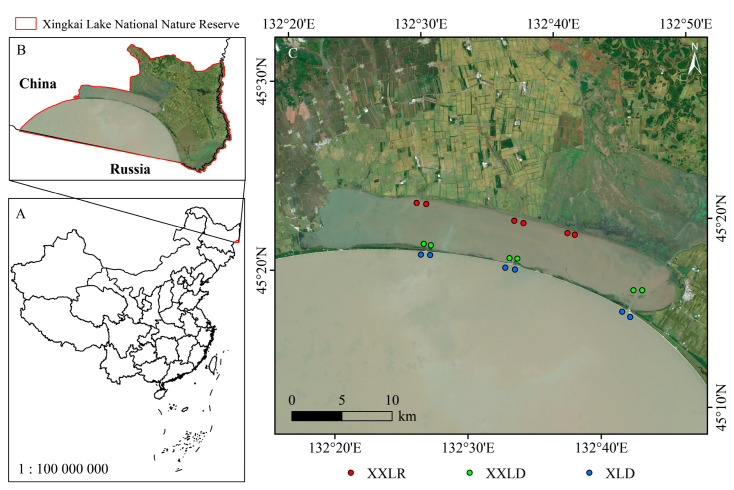
Sampling map. The study area was divided into three regions: the inlet of the river upstream of XXL (XXLR), near the dam of XXL (XXLD) and XL (XLD). (**A**) Map of China, (**B**) Location of Xingkai Lake National Nature Reserve, (**C**) Distribution map of sampling sites.

Xingkai Lake (XL), extending from 131°58′ E to 133°07′ E and 45°01′ N to 45°34′ N, is located at the border between China and Russia (Figure 1). Xiao Xingkai Lake (XXL) is an inland lake adjacent to XL and connected to XL through dams. The climate there is a typical temperate continental monsoon climate, and the average annual temperature is as low as 3 °C [21]. The Xingkai Lake basin has diverse habitats surrounded by a large number of wetlands, swamps, and farmland; moreover, XL plays a significant role in water conservation, groundwater recharge, and climate regulation [22]. However, the rising agriculture and tourism around have resulted in severe water pollution and mild eutrophication of XL.

The hydrological conditions in the Xingkai Lake Basin are complex. The upper reaches of XXL are fed by agricultural irrigation rivers, and the lower reaches are connected to XXL through dams. Hydrological connectivity plays a crucial role in the biodynamic and physicochemical variables of connected lakes. In view of this, we divided this area into three regions according to their special hydrological conditions (Figure 1C): the river inlet upstream of XXL (XXLR), near the dam of XXL (XXLD) and XL (XLD). Sediment samples were collected in the XXL and XL from 8 January to 17 January 2022. During the sampling period, the weather was sunny and the temperature was about negative 30 °C, resulting in the entire lake surface being frozen. Thus, we used ice drills to break the ice, and intact sediment cores were collected using a UWITEC sediment corer along a depth gradient. The upper layer of 0–10 cm was retrieved from three replicate cores at each position and was then uniformly blended; ultimately, one sample was obtained from each sampling position. In total, eighteen sediment samples were collected and sent to the laboratory for further analysis.

### 2.2. Sediment Property Analysis

All sediment samples were freeze-dried to constant weights, and roots and stones were removed. Subsequently, these samples were softly grinded in a mortar and then were passed through a 2 mm sieve. Sediment pH was measured in 2.5:1 mixtures of deionized water and soil using a pH meter [23]. Total carbon (TC), total nitrogen (TN), and total sulfur (TS) were measured using an elemental analyzer (Vario EL III Elementer, Germany). Dissolved organic carbon (DOC) was extracted using the soil-to-distilled water in a ratio of 1:5 (*w*/*v*) and finally determined with a TOC analyzer. For specific operation steps, please refer to [24]. Labile Organic Carbon (LOC) was measured based on potassium permanganate oxidation method [25,26]. Total sediment phosphorus (TP) content was determined using perchloric acid digestion and a UV-2550 spectrophotometer (Shimadzu, Kyoto, Japan). The molybdenum blue colorimetric method was employed to determine the concentration of available phosphorus (AP).

### 2.3. DNA Extraction and Sequence Processing

Sediment DNA was extracted from 0.5 g wet sediment using a Power Soil DNA Isolation Kit (MO BIO Laboratories, Inc., Carlsbad, CA, USA). The extracted DNA was then preserved in −80 °C until the next step. Specific primer pairs (341F, 5′-CCTAYGGGRBGCASCAG-3′; 806R, 5′-GGACTACNNGGGTATCTAAT-3′) combined with the adapter sequence and barcode sequence were used to amplify the distinct regions (V3–V4) of the bacterial 16S rRNA genes. All PCR reactions were performed with 15 μL of Phusion^®^ High Fidelity PCR Master Mix (New England Biolabs, Ipswich, MA, USA), 0.2 μM of forward and reverse primers, and approximately 10 ng of template DNA. Thermal cycling comprised 30 cycles of initial denaturation at 98 °C for 1 min, followed by denaturation at 98 °C for 10 s, annealing at 50 °C for 30 s, and elongation at 72 °C for 30 s, and finally 72 °C for 5 min. Sequencing was employed on an Illumina NovaSeq platform (Novogene Co., LTD, Beijing, China) and 250 bp paired-end reads were generated.

The 16S rRNA sequencing data were analyzed using QIIME 2 (Quantitative Insights into Microbial Ecology, https://qiime2.org (accessed on 7 July 2022)). Paired-end reads were merged using FLASH and were denoised using DADA2 in QIIME 2 [27,28]. Sequences were classified using the SILVA database (v.138). The amplicon sequence variants (ASVs) were defined using a 97% similarity cutoff. The percentage of classified taxa is 88.18%. All the sequencing data of sediment bacterial community used in this study are available in the NCBI Sequence Read Archive by accession no. PRJNA935412.

### 2.4. Community Assembly Processes

Stegen proposed a framework to assess the effects of ecological drift, selection, and dispersal in regulating community assembly using null models [29,30]. Here, the top 1000 ASVs were selected to operate these models, which avoided the impacts of sequencing errors, including singletons, and hardly influenced the results [31]. First, we quantified the observed beta-mean-nearest taxon distance (βMNTD) to estimate the phylogenetic turnover using the comdstnt function in “picante” of R package [32]. Subsequently, the null distribution of βMNTD was created by randomizing the ASVs of the phylogenetic tree 999 times. Finally, the beta-nearest taxon index (βNTI), which is the standard deviation between the observed βMNTD values and the mean of the null βMNTD values was computed to indicate the ecological processes in specific communities [33]. βNTI values > 2 suggest that phylogenetic turnover is significantly greater than expected and is controlled by heterogeneous selection (variable selection); βNTI values < −2 mean that phylogenetic turnover is significantly less than expected and homogeneous selection dominants the community assembly. Simultaneously, |βNTI| values < 2 demonstrate that stochastic processes primarily regulate the phylogenetic turnover, and the Raup-Crick metric based on Bray-Curtis (RC_bray_) needs to be further calculated to disentangle explicit processes. The RC_bray_ is calculated as the difference between the observed Bray-Curtis dissimilarity and its null distribution. RC_bray_ > +0.95 reveals the dispersal limitation; RC_bray_ < −0.95 represents the homogenizing dispersal and |RC_bray_| < 0.95 refers to “undominated” processes including drift, dispersal, diversification, and weak selection [30,34,35]. All the analyses were performed in R v.4.0.5 with the package “picante”; detailed information could be located in the Supplementary Dcument of [30].

### 2.5. Co-Occurrence Network Construction

To evaluate the species coexistence of microbial communities in sediments, the metacommunity cooccurrence networks of all three regions (XXLR, XXLD, and XLD) were constructed. The ASVs selected were the same as we used in community assembly. Robust correlations with Spearman’s correlation coefficient (ρ) > 0.8 and *p* < 0.01 (*p*-values were adjusted using the Bonferroni procedure) were remained to construct the network edges [36,37]. Afterwards, we extracted the subnetwork topological features from different regions for subsequent analysis. All cooccurrence network features among the bacterial communities were analyzed in R v.4.0.5 using Spearman’s correlation via the R package “igraph”.

### 2.6. Statistical Analysis

One-way ANOVA was employed to compare the differences in sediment physical and chemical properties using SPSS (version 23.0, IBM Crop., Armonk, NY, USA). Non-metric multi-dimensional scaling (NMDS) analysis based on Bray–Curtis dissimilarity and analysis of similarities (ANOSIM) were conducted to evaluate the complexity of the community composition and compare the differences between sampling regions. Both NMDS and ANOSIM were performed in R v.4.0.5 using the “vegan” package. The α-diversity was calculated using the “picante” package. In this study, β-diversity was quantified as the average distance of group members to the group centroid in the vegan package based on the Bray–Curtis distances [38]. Then, we investigated the mechanisms involved in the assembly processes of sediment bacterial communities and their network co-occurrence patterns (as mentioned above). In addition, Mantel test was executed to evaluate the relationships between the βNTI of the bacterial community and sediment environmental properties. The Levin’s niche breadth of the species was calculated using the “spa” package to demonstrate the community sensitivity to the sediment environment [39]; moreover, the niche width influences the relative importance of deterministic processes and stochastic processes [40]. We also assessed the relationships among the sediment properties, network features and community diversity employing Spearman’s correlation and Mantel test. To understand the drivers of microbial community structure and diversity in different regions, we further performed the partial least squares (PLS) path analysis using the “plspm” package in R.

## 3. Results

### 3.1. Sediment Chemical Properties

The sediment physicochemical properties varied among the different sampling regions (Figure S1a). Specifically, TC, TN, TP, DOC, and LOC present similar trends, with significant enrichment in region XXLD. Among them, TC (%) of XXLR and XLD was only 0.653 ± 0.086 and 0.478 ± 0.065, TN (%) was only 0.065 ± 0.009 and 0.062 ± 0.008, and TP (mg/kg) was only 329.180 ± 35.425 and 348.993 ± 16.469, respectively. Interestingly, the contents of TC, TN, and TP reached 3.305 ± 0.801, 0.245 ± 0.058, and 523.597 ± 101.785 in XLD, respectively. In addition, DOC and LOC were also significantly enriched in the XXLD region (174.83 ± 31.640 mg/kg and 7.697 ± 2.078 g/kg, respectively). In addition, higher pH was shown in region XLD; TS showed lower levels in region XXLR. Except for total sulfur, other environmental factors showed a significant positive or negative correlation (Figure S1b).

### 3.2. Bacterial Community Composition and Diversity

**Figure 2:**
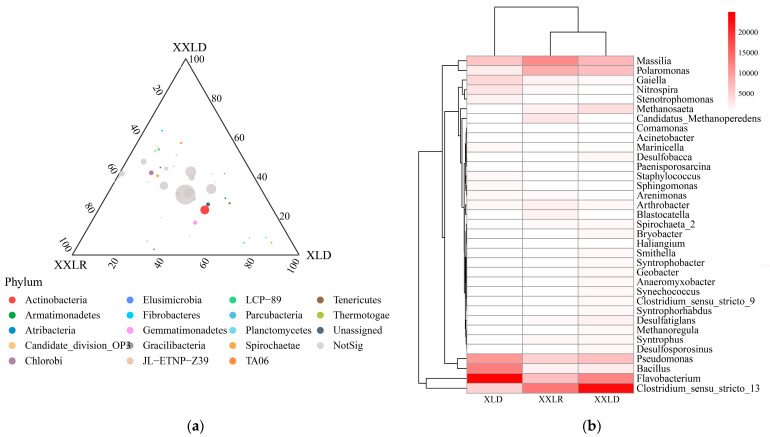
Sediment bacterial community composition among different regions. (**a**) Ternary plots of ASVs across three sampling regions. The size of each point represents the relative abundance of ASV. The position is determined by the contribution of three sampling regions to the total relative abundance, proximity to that vertex indicates enrichment of that ASV in that region. The colors of the circles correspond to different phyla. Grey circles indicate ASVs with no significant differences in abundance. (**b**) Heatmap displaying the absolute abundance of the top 35 genera. XXLR: the inlet of the river upstream of XXL, XXLD, and XLD: near the dam of XXL and XL.

The sediment bacterial community composition exhibited a distinct variation among different sampling regions (Figure 2 and Figure S2). As shown in Figure S2a, more than 7000 ASVs were detected and over 6000 shared bacterial ASVs were observed in all regions. There were 44 bacterial phyla identified within all samples. The top 10 phyla were shown in Figure S2b, and Proteobacteria (39–45%) was the most abundant phyla in all regions, followed by Firmicutes (8–14%) and Bacteroidetes (6–12%). The relative abundances of 17 different identified-bacterial phyla showed significant differences among different regions by comparing the abundance of all bacterial phyla (Figure 2a). This indicated that these top-abundance bacterial phyla showed little differences while these low-abundance phyla exposed significant differences, which possibly caused a different bacterial community structure and diversity. Moreover, the top 35 bacterial genera also present a significant difference (Figure 2b). For example, Clostridium_sensu_stricto_13 is more abundant in region XXLD while Flavobacterium is more abundant in region XLD.

**Figure 3:**
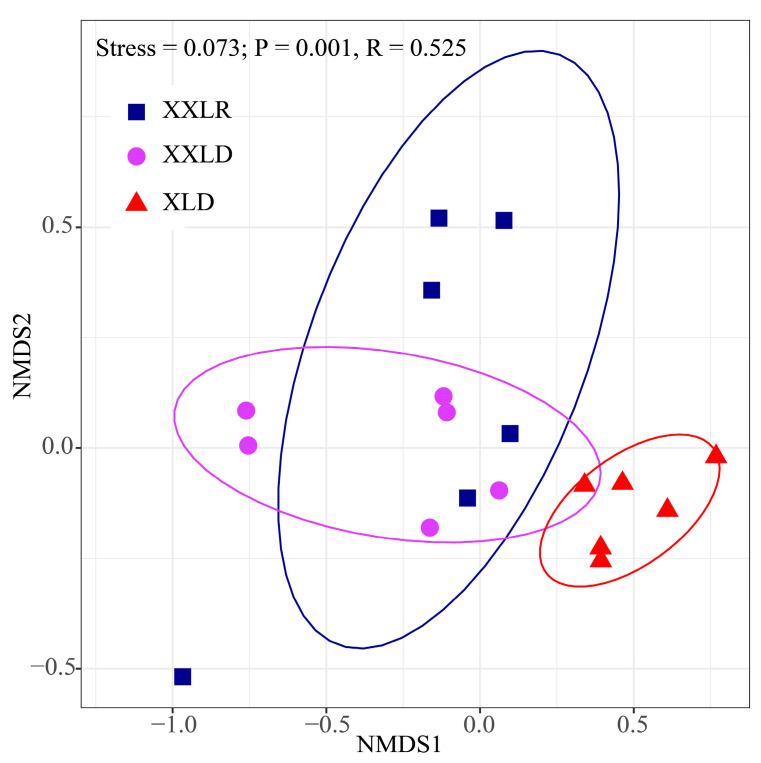
Nonmetric multi-dimensional scaling (NMDS) analysis based on Bray-Curtis dissimilarity at the ASV level of sediment bacteria. Significant differences among plant species and all radial layers were tested by analysis of similarities (ANOSIM) analysis. XXLR: the inlet of the river upstream of XXL, XXLD, and XLD: near the dam of XXL and XL.

The α-diversity indices of the bacterial community did not show significant differences (Figure S3a). The nonmetric multidimensional scaling (NMDS) analysis of Bray–Curtis distances at ASV level was performed to investigate the differences of sediment bacterial community structures between regions. According to NMDS (Stress = 0.073) and ANOSIM (R = 0.525, *p* = 0.001) analysis, these sampling regions contained different bacterial community structures (Figure 3). In addition, we revealed β-diversity (Wilcox test, *p* < 0.05) indicated significant differences among different sampling regions (Figure S3b).

### 3.3. Bacterial Community Assembly Processes and Co-Occurrence Network

**Figure 4:**
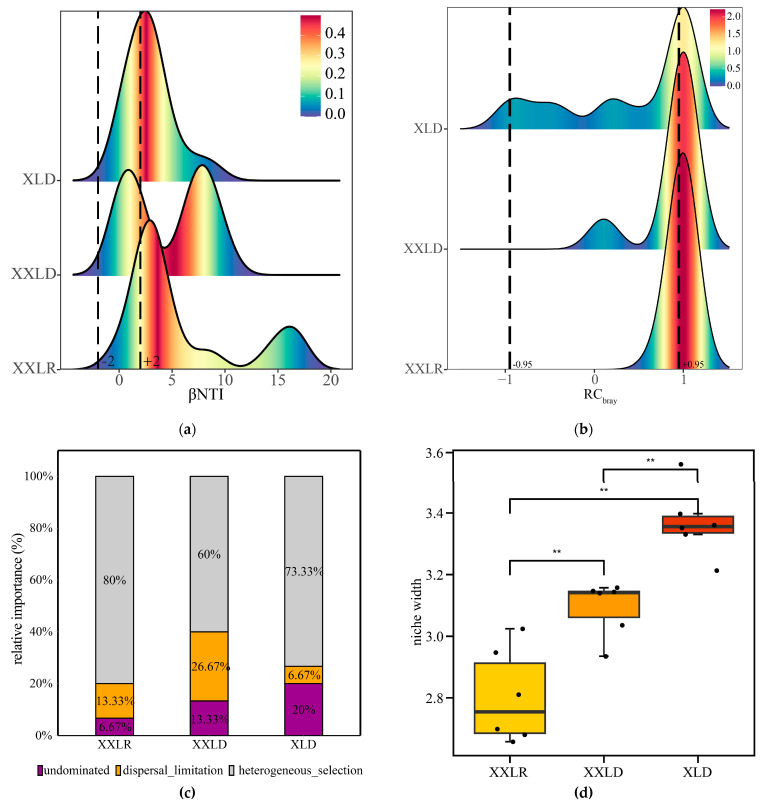
Distribution of phylogenetic turnover (βNTI; (**a**)) and taxonomic turnover (RC_bray_; (**b**)) and the percentages of the three assembly processes (**c**) and niche width (**d**). The vertical dashed lines mark the positions of −2 and 2 in panel a and −0.95 and 0.95 in (**b**). The percentages on the bar graph indicate the relative importance of different assembly processes (**c**). Significant differences of niche width across the regions were indicated by ** *p* < 0.01 (**d**). XXLR: the inlet of the river upstream of XXL, XXLD, and XLD: near the dam of XXL and XL.

The assembly process of the microbial community was assessed by the phylogenetic turnover of the bacterial communities, which was primarily evaluated through the β nearest taxon indices (βNTI); the main results are presented in Figure 4. We found that the majority of absolute βNTI values (60–80%) were above 2 in all three regions, indicating the dominant role of deterministic processes (Figure 4a). The RC_bray_ index mainly distributed between −0.95 and 1 (Figure 4b). Specifically, heterogeneous selection played a leading role in the assembly of bacterial communities in the sampling regions; in addition, dispersal limitation was more important for bacterial community assembly in the regions XXLR and XXLD while the undominated processes (e.g., ecological drift) were more important in region XLD (Figure 4c). Moreover, we found that bacterial communities in region XLD showed higher ecological niche breadth (Figure 4d). We also probed the relationships between sediment environmental factors and βNTI using Mantel test (Table S1). The results certified that TS were significantly associated with βNTI (*p* < 0.05). This suggested that TS affected sediment bacterial community structure.

**Figure 5:**
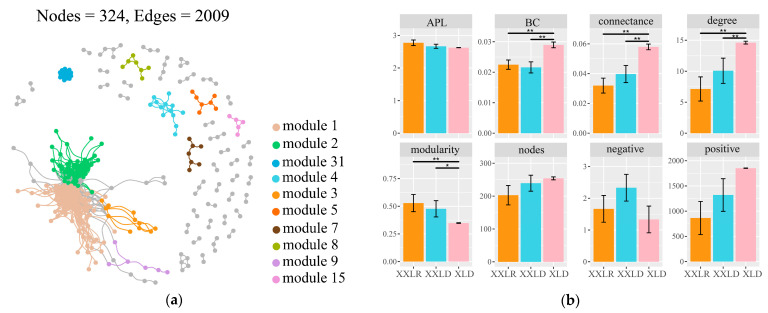
Co-occurrence networks and the subnetwork topological features. Visualization of constructed co-occurrence networks among all samples in different regions (**a**). Top 10 modules were shown in different colors, and smaller modules were shown in grey. Comparisons of subnetwork topological features in different regions (**b**). Data were shown as the mean ± standard error (n = 6). The significance was analyzed based on the Wilcox test: * *p* < 0.05, ** *p* < 0.01. APL: average path length; BC: betweenness centralization. XXLR: the inlet of the river upstream of XXL, XXLD, and XLD: near the dam of XXL and XL.

Co-occurrence networks were constructed to investigate the biotic interactions across microbial communities in different sediment environments (Figure 5). Overall, the global network obtained 324 nodes and 2009 edges, and the top ten modules are represented with different colors (Figure 5a). We then extracted the subnetwork topological features (Figure 5b). The higher degree of modularity in region XXLR and XXLD implied stronger interactions among species within the same module. The majority of the microbes in all regions were positively correlated, demonstrating a strong co-occurrence pattern in bacterial communities. The average degree, connectedness, and betweenness centralization were significantly higher in region XLD, suggesting a more complex interspecific interaction.

### 3.4. Factors Shaping the Community Diversity in Sediment

**Figure 6:**
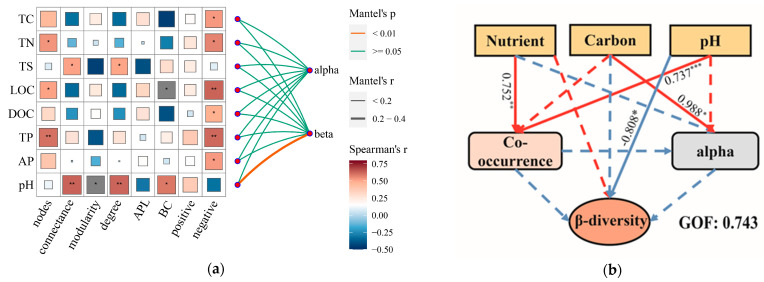
Mantel test analysis and PLS paths showing the relationships of sediment environmental variables to network features, α- and β-diversity. (**a**) Mantel test analysis. Spearman correlation was used between environmental variables and network features. APL: average path length; BC: betweenness centralization. (**b**) PLS paths analysis. Red and blue lines represent positive and negative path coefficients, respectively. The path coefficients represented the “strength and direction” of the relationships between the response and the predictors. Dotted lines indicated the path coefficients without statistical significance. Ellipses and rectangles represent latent variables and observed variables. Significant differences were indicated by * *p* < 0.05, ** *p* < 0.01, *** *p* < 0.001. GOF: goodness of fit. XXLR: the inlet of the river upstream of XXL, XXLD, and XLD: near the dam of XXL and XL.

Considering the roles of biotic and abiotic factors, we conducted partial least squares (PLS) path analysis to explore how these factors determined microbe community diversity in the sediment. First, we examined the correlation between environmental factors and network topological features and community diversity (Figure 6). The results revealed that nutrient elements, including TN, TP, and TS, as well as the carbon components, were significantly correlated with the network features. Furthermore, pH showed a significant correlation with beta diversity. Then, we further employed PLS analysis with nutrient composition (TN, TP, TS), carbon composition (TC, LOC, DOC), and pH as environmental variables, combined with network topological features (nodes, connectedness, degree, betweenness centralization) to illuminate the direct and indirect contributions of biotic and abiotic elements to the impact of community diversity. The ultimate PLS analysis explicated 82.5%, 62.8%, and 69.0% of the variation in network characteristics and community diversity (α and β), respectively. This indicated that sediment physicochemical properties could alter network steady-state and determine the bacterial community diversity.

## 4. Discussion

### 4.1. Variation Characteristics of Sediment Properties

In our study, we noticed the enrichment of nutrients and carbon at the downstream dam of Xiao Xingkai Lake (region XXLD). This may be due to the reason that despite the input of nutrient-rich water sources for farmland irrigation and drainage, the upstream impact of water flow is not conducive to the deposition of these elements. Alterations in natural flow regimes cause changes in upstream and downstream hydrological and ecological conditions [41,42]. In the lower reaches of the Xiao Xingkai Lake (region XXLD), the existence of the dam reduced the normal water exchange gradient with the Xingkai Lakes, which promoted the increase of carbon content and facilitated the accumulation of nutrient elements. Previous studies have also confirmed this explanation: the presence of dams dramatically altered important habitat properties such as dissolved oxygen, electric conductivity, water content, total nitrogen, and total phosphorus in sediment [43]. In addition, dam construction changed the nitrogen transport process consequently prompting a dual impact on the regional water environment and hydrodynamic disturbance made the endogenous nitrogen release load in sediments the second largest source of nitrogen in the estuary, which might be an important factor in accelerating estuarine algal blooms and habitat degradation, and the accumulation of P in water through river flows probably leads to eutrophication [44,45].

Overall, building dams alters biogeochemical cycles, such as interrupting the flow of organic carbon, altering nutrient balance, and altering oxygen and thermal conditions. The consequences of this may not be immediately apparent. Due to the complexity and uniqueness of aquatic ecosystems, it is difficult to make precise predictions about the impact of a particular dam. Due to the inflow of massive agricultural water in the Xingkai Lake Basin, nutrients have gradually accumulated and enriched in the XXL. Cyanobacteria can thrive in conditions of high nutrient concentration and low water flow [46]. Therefore, it can be inferred that if no improvement measures are taken, the water quality and algal bloom problems in Xingkai Lake will become more and more serious in the future.

### 4.2. Community Assembly Process and Microbial Interactions

Assessing the relative significance of deterministic and stochastic processes on community assembly is important for identifying factors affecting community composition [29]. We employed two null models (βNTI and RC_bray_) to quantify different community assembly processes, finding that deterministic processes dominated within all the regions. For bacterial communities, stochastic and deterministic processes play important roles in community assembly, with determinism sometimes dominating and stochasticity sometimes more important [47,48]. Furthermore, a greater impact of the deterministic process on bacterial community assembly appeared in human-affected areas with certain environmental variables rather than in the natural environment [29,49]. For example, temperature is one of the main factors regulating the balance of stochastic and deterministic assembly processes in hot spring sediment communities, with differences in temperature increasing the contribution of determinism [50]; the increasing levels of eutrophication likely enhance the deterministic process of bacterial community assembly [51]. In addition, a dominant role of homogeneous selection in microbial community assembly processes has been reported in the Three Gorges Reservoir sediments [52]; stochasticity regulates the assembly processes of bacterial communities in the sediment of shrimp culture ponds [53]. One potential explanation for this discrepancy in the prevalent community assembly processes could attribute to differences in the studied spatial scales [54,55]. To gain a basic mechanistic understanding of microbial ecology [56], it is essential to determine the environmental factors that shape the processes of microbial community assembly. Recent studies have revealed that pH [57], organic carbon content [58], salinity [59], available sulfur [60], and TN [33] are important factors influencing bacterial community assembly processes in different habitats. In our study, we found that bacterial community βNTI was significantly correlated with sediment total sulfur, with higher TS content promoting deterministic ratios. Related studies have informed that the sulfate content in the sediment determines the assembly process [61]. Sulfide toxicity to microbes and sediment acidification and salinization may partially illustrate the effect of sediment sulfate concentration in regulating bacterial community assembly [62,63,64]. However, our study does not figure out which part of sulfur regulates the assembly of bacterial communities, and a future study should pay more attention. In addition, previous studies have found that low soil nutrient conditions contribute to deterministic assembly processes while higher nutrient conditions increase stochastic assembly processes [58,65,66], which could be a potential reason for the relatively high stochastic process in XXLD region. In addition, generalists with wider habitats are less affected by environmental factors [40,67,68]. The community assembly in our study was dominated by heterogeneous selection due to their narrow niche width, implying a low tolerance to environmental disturbances.

Microbial co-occurrence networks provide important insights into microbial community properties. Our results showed that in different regions, the bacterial community exhibited different degrees of network properties, among which region XLD exhibited relatively stronger network interactions. Different environmental factors had a significant impact on these network properties (Figure 6). Relevant studies have indicated that pH is a dominant factor in bacterial network formation, with higher connectivity and stability appearing in soils characterized by neutral pH than in acidic or alkaline soils within diazotrophic communities [69,70]. This finding also supports our results. Furthermore, we found that more than one environmental factor (TN, TP, and TS), rather than only pH, caused this difference in network complexity.

### 4.3. Structure and Diversity of Sediment Bacterial Communities

In microbial ecology, the mechanisms of community diversity have been extensively explored [35]. Here, we investigated the structure and diversity of sediment bacterial communities in different regions of Xingkai Lake. The results of the community composition showed that Proteobacteria, Firmicutes, Bacteroidetes, Acidobacteria, and Actinobacteria were the most abundant phyla in different regions. The total relative abundance of these phyla exceeded 70% of the entire community, among which Proteobacteria accounted for 39–45%. These bacteria widely exist in various habitats and display rich metabolic diversity. Importantly, they play a significant role in sediment element circulation, including C, N, S, and Fe [71]. Our result is consistent with many previous studies [8,51,72]. Within Proteobacteria, Gammaproteobacteria, as the most representative Proteobacteria in eutrophic lakes [73], is one of the prevalent groups in Xingkai Lake sediments. Firmicutes exist in various habitats in Xingkai Lake and some genera can form spores, thus surviving under extreme environmental conditions. Many studies support the result that Firmicutes was identified as the second most abundant phyla in sediment bacteria [74,75]. In lake sediments contaminated with metals, sequences associated with Firmicutes may account for nearly 50% of all bacteria, and high levels of trace metal contamination in lakes could be responsible for the abundance of endospore-forming Firmicutes [76]. In the future, we should pay more attention to the relationship between the changes in the abundance of Firmicutes and the possible pollution of Xingkai Lake. In addition, Firmicutes prefer eutrophic conditions, which may be another reason for the prevalence of Firmicutes [77]. Bacteroides are widespread in soil, sediment, and seawater [78]. Some genera of Bacteroides are pathogenic, whereas most are commensal bacteria that are highly adapted to the gastrointestinal tract [79]. This group also comprises the most abundant rhizosphere bacteria. Certain classes of Firmicutes and Bacteroides play key roles in the decomposition and fermentation of organic matter in sediments [11,80,81]. Previous studies have found that pH has a significant effect on the abundance of Acidobacteria; their abundance especially increases when the pH is lower than 5.5 [82].

Microbial community diversity and phylogeny differences likely result from many different factors, the most critical of which are those characteristics that define the physical and chemical environments of communities [72]. In sediment ecosystems, chemical gradients and physical stratification contribute to the creation and maintenance of high levels of diversity among and within bacterial communities [83,84]. Environmental factors are crucial in determining bacterial community composition. In our study, we revealed that carbon significantly affect the bacterial α-diversity, which is consistent with previous studies [85,86]. Furthermore, we disclose that pH greatly influences the sediment bacterial community diversity. Many studies have reported the key role of pH in regulating variation in bacterial community diversity [57,74,87,88]. We can explain the important role of pH in shaping community diversity from two aspects: First, soil pH may not directly change the bacterial community structure but may work as a comprehensive variable. The pH value directly or indirectly alters the physicochemical properties of the sediment, which further changed the bacterial community structure and diversity. Second, pH affects bacterial enzymes to determine bacterial metabolism, growth, and reproduction [89], ultimately affecting the structure and diversity of bacterial communities. In addition, trophic status plays an important role in driving lake sediment microbial communities [90], which should be focused on in Xingkai Lake in the future study.

## 5. Conclusions

In our study, we investigated the bacterial community structure and diversity characteristics in the sediments of Xiao Xingkai Lake and Xingkai Lake and how these characteristics responded to changes in the sediment environment. We found that the sediments in the Xingkai Lake Basin exhibited different physicochemical properties under different hydrological conditions, and the impact of the dam significantly lead to the enrichment of carbon, nitrogen, and phosphorus. Proteobacteria, Firmicutes, and Bacteroidetes were the prevalent bacterial phyla in sediments. Variations in nutrient loads (TN, TP, TS), carbon (TC, LOC, DOC) and pH lead to changes in sediment microbes, with pH being the most important factor determining differences in sediment bacterial community diversity across regions with different hydrological conditions. In conclusion, our findings have important implications for a comprehensive description of the biogeochemical processes of bacteria in sediments and ecological restoration and construction in the Xingkai Lake area. In the future, we should also focus on the impact of lake eutrophication on sediments and microbial communities in Xingkai Lake.

## Data Availability

Research data was presented in the tables and figures in the main text of the article.

## Supplementary Materials

The following supporting information can be downloaded at: https://www.mdpi.com/article/10.3390/microorganisms11030676/s1, Figure S1: Differences and correlations of sediment physicochemical factors in different regions of Xingkai Lake; Figure S2: Microbial community composition and structure; Figure S3: Bacterial community α- and β-diversity; Table S1: Relationship between sediment physicochemical properties and phylogenetic turnover of bacteria.
