# Supplementary material for: pH Drives Differences in Bacterial Community β-Diversity in Hydrologically Connected Lake Sediments
Source: Microorganisms. 2023 Mar 7;11(3):676. doi: 10.3390/microorganisms11030676 (PMC10056738; doi:10.3390/microorganisms11030676)
Supplement: Supplementary file 1 [file microorganisms-11-00676-s001.zip › microorganisms-2214956-supplementary.pdf]

pH drives differences in bacterial community β-diversity in hydrologically connected lake sediments

Haiguang Pu [1, 2], Yuxiang Yuan [1], Lei Qin[1], Xiaohui Liu [1,*]

1 Key Laboratory of Wetland Ecology and Environment, Northeast Institute of Geography and Agroecology, Chinese Academy of Sciences, Changchun, China
2 University of Chinese Academy of Sciences, Beijing, China
* Correspondence: liuxh2752@126.com.

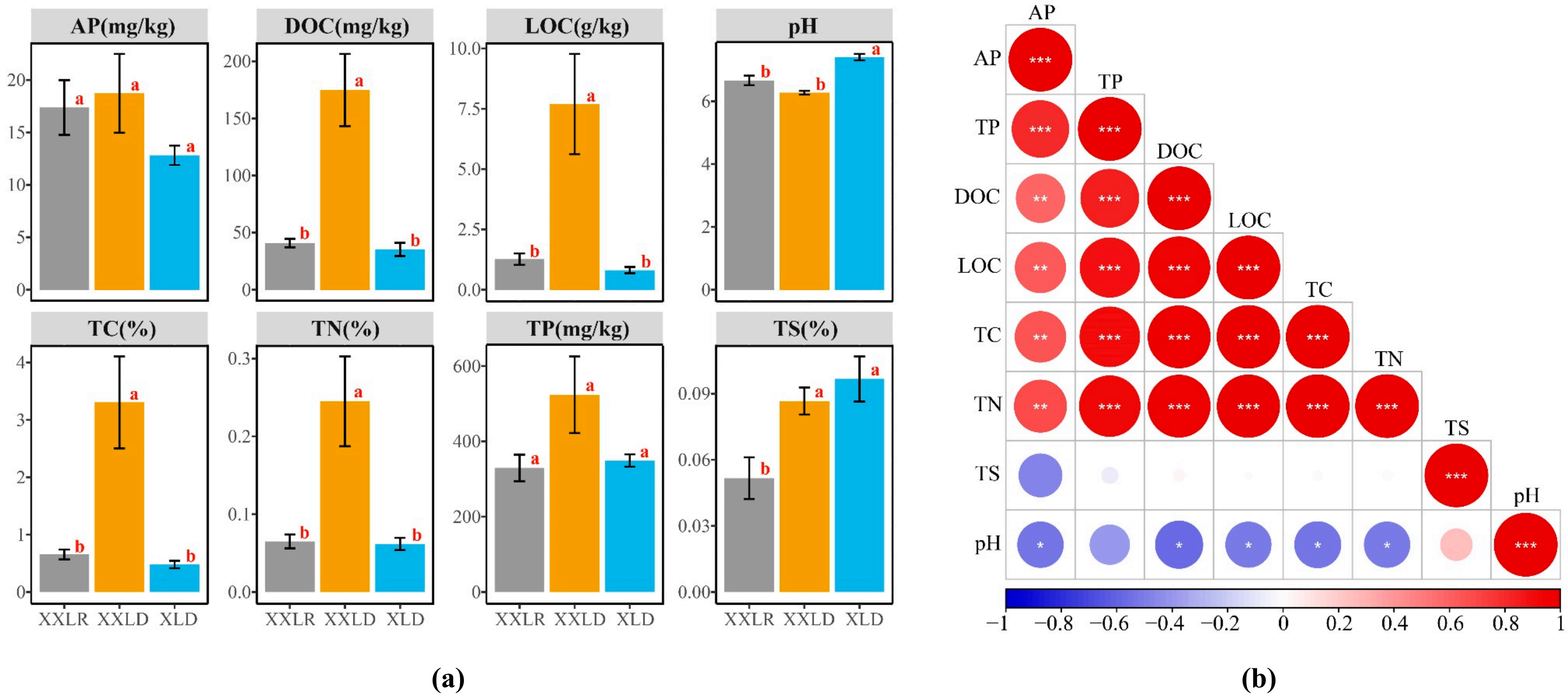

Figure S1. Differences and correlations of sediment physicochemical factors in different regions of Xingkai Lake. Comparisons of sediment physicochemical factors used one-way ANOVA analyses (a). Lowercase letters indicate significant differences (Turkey test, $p < 0.05$). Data were shown as the mean ± standard error ($n = 6$). Correlation matrix graph indicating the correlation between sediment physicochemical factors (b). XXLR: the inlet of the river upstream of XXL, XXLD and XLD: near the dam of XXL and XL.

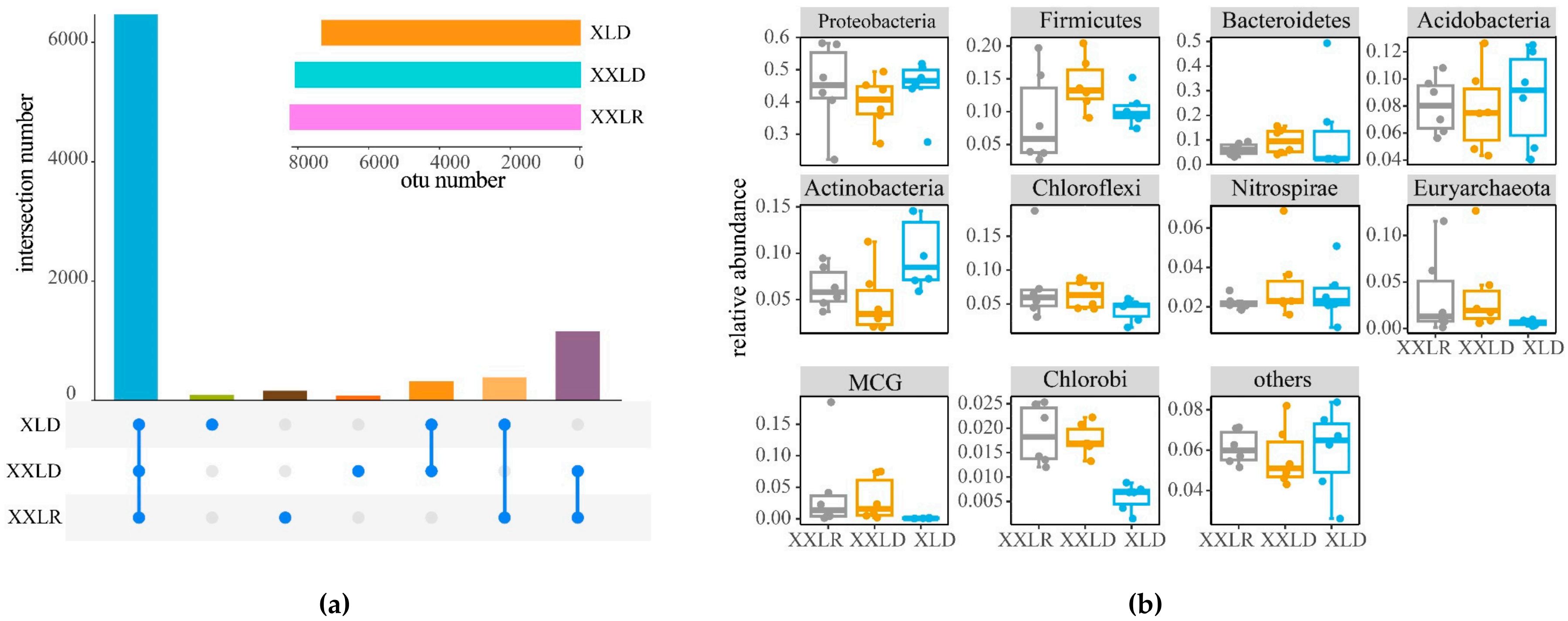

Figure S2. Microbial community composition and structure. (a) Histogram of the shared OTUs. The horizontal bar graph in the upper right corner indicates the total number of OTUs. (b) Boxplot of the top ten bacterial phyla in relative abundance. MCG: Miscellaneous_Crenarchaeotic_Group, XXLR: the inlet of the river upstream of XXL, XXLD and XLD: near the dam of XXL and XL.

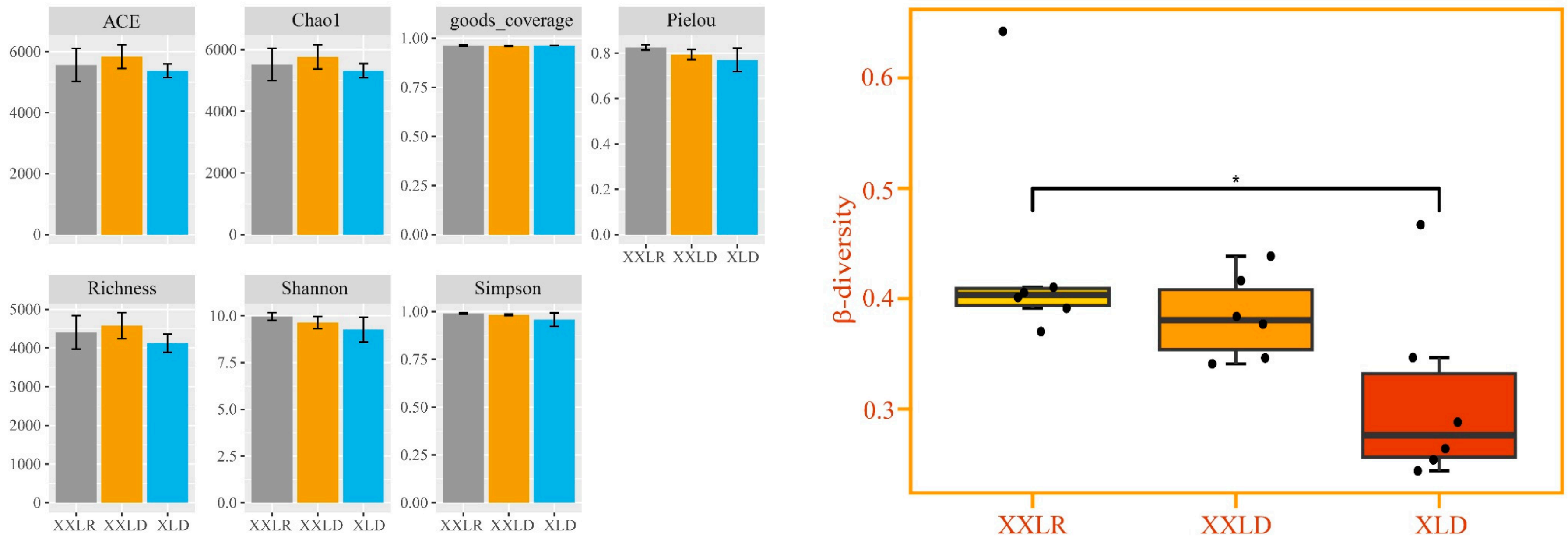

(a) **(b)**

Figure S3. Bacterial community α- and β-diversity. (a) Histogram of alpha diversity index. Data were shown as the mean ± standard error (n = 6). (b) Boxplot of β-diversity. β-diversity was calculated as the average distance of group members to the group centroid based on Bray-Curtis dissimilarity. Significant differences of β-diversity across the regions were indicated by $*p < 0.05$ (c).

Table S1. Relationship between sediment physicochemical properties and phylogenetic turnover of bacteria (Mantel test).

	TC	TN	TP	AP	TS	LOC	DOC	pH
r	-0.134	-0.182	0.101	0.102	0.231	-0.139	-0.078	0.025
p	0.923	0.981	0.147	0.128	**0.017**	0.928	0.758	0.338
